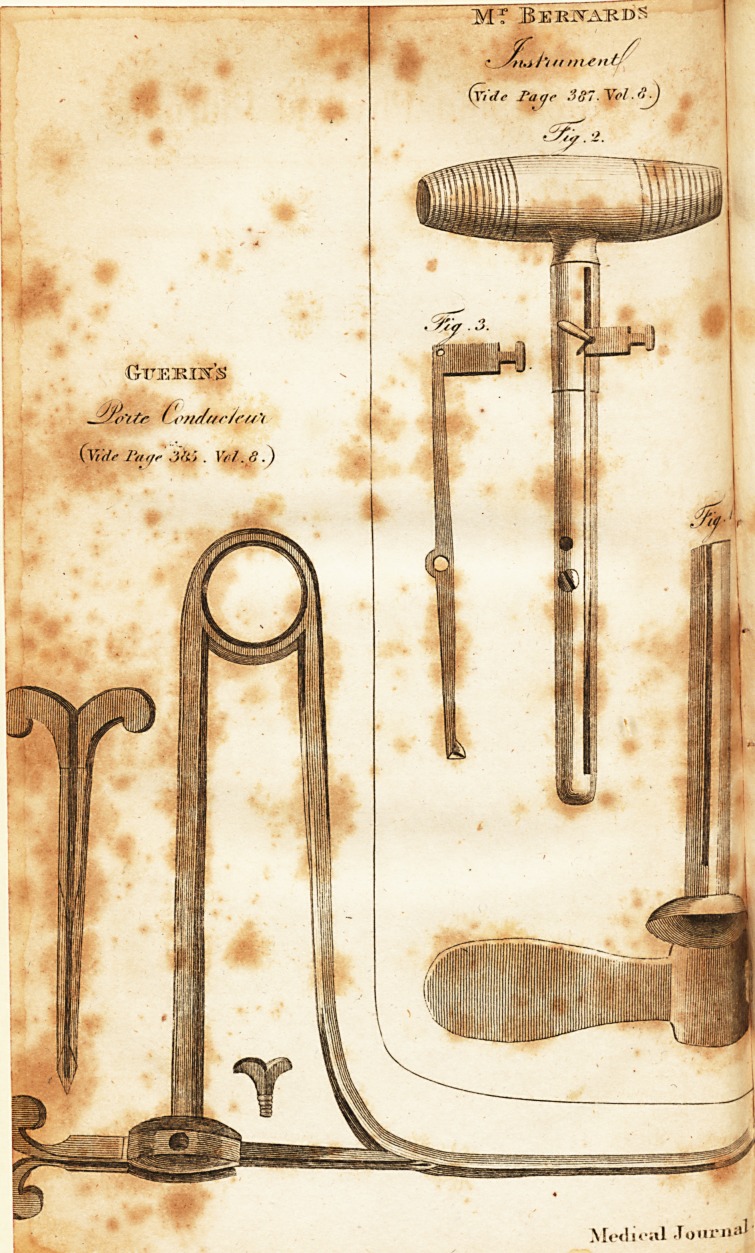# A Description of a New Instrument for Performing the Operation of Lithotomy, Invented by Citizen Guerin
*We are indebted for this communication to Dr. Ehlers, of Altona, who on his return from Paris happened to pass through Gottingen, where he kindly permitted Dr. Noehden to make a drawing of this new instrument.


**Published:** 1802-11-01

**Authors:** 


### Medical and Phyfical Journal.

VOL. VIII.] NOVEMBER 1, 1802. [NO. XLV.

*Printed for R. Phillips, by W. Thorne, Red Lion Court, Fleet Street, London.*

### *A* DESCRIPTION *of a* NEW INSTRUMENT *for performing the* OPERATION *of* LITHOTOMY, *invented by Citizen* GUERIN.*

[ With an Engraving. ]

THE incifion of the canalis urethræ, an effential part of the lateral operation, is often attended with many difficulties; and it is on this account, that fome operators make it too high for the fake of finding more eafily the groove of the ftaff; it is, however, frequently the cafe, that for fear of having miffed the the groove, the knife, (lithotome) is withdrawn, in order to give it the proper direction, or that it is introduced a fecond time, if the incifion has not been made large enough; in both which cafes it is not uncommon that a fecond incifion is made along the firft, by which the canalis urethræ is confiderably injured. This accident, which particularly happens to beginners, is extremely hurtful, by increafing the difficulty of introducing the inftrument, which is to cut the collum veficæ into the bladder. There is befides, much danger of the point of the inftrument getting out of the groove of the ftaff, and of its fliding between the inteftinum rectum and the bladder; a miftake which may prove of very fatal confequences.

For preventing this danger, and for rendering this part of the lateral operation lefs difficult, Citizen Guerin has added to the common canaliculated probe or ftaff, a part which he calls *porte-conducteur*. It proceeds from the upper extremity of that probe; and forming an arch, it leaves a fufficient fpace for the penis and fcrotum; it then goes downwards in a ftraight line almoft to the curvature of the probe, whence the inci-

* We are indebted for this communication to Dr. Ehlers, of Altona, who on his return from Paris happened to pafs through Gottingen, where he kindly permitted Dr. Noehden to make a drawing of this new inftrument.

ſion is begun. There it ends in an oval caſe, perforated with a round hole of a line and a half in diameter, through which is to paſs a trocar of a proportionable ſize, which being fixed in the caſe by means of a ſcrew, is intended to ſerve as a conductor. The caſe itſelf has, underneath, a chink from one end to the other, one ſide of which is perpendicular, while the other is a little inclined towards the right, forming with the former an angle of about 45°, the baſis of which is towards the outſide. The chink exactly correſponds with the groove of the trocar, and is intended to receive and to conduct the Lithotome; the trocar is three inches and a half long, and has a groove which terminates in a triangular point, which exactly fits the groove of the ſtaff; the oppoſite end has a handle in the ſhape of a croſs, with which the proper direction may be given to it. The inſtrument having been introduced into the bladder, after the trocar has been withdrawn to its point, and fixed in the caſe by means of the ſcrew, is lifted up under the angulus pubis, as in the manner deſcribed by Cheſelden; the trocar is now looſened and puſhed forward againſt the perinæum, which having pierced, it paſſes to the groove of the ſtaff, with which it now forms one continuity, and thus fixes the neck of the bladder. When the trocar is faſtened by the ſcrew in ſuch a manner that its groove is exactly turned towards the chink of the caſe, the blade of the lithotome ought to be taken between the fore-finger, which is laid on its inferior edge, the thumb on its exterior ſide, and the middle finger on its interior ſide, while its handle lies in the palm of the hand. The point of the blade being brought into the groove of the trocar is puſhed along it, and thus conducted into the groove of the ſtaff.

The advantages of this proceeding are very obvious, and may be mentioned in a few words. They conſiſt in cutting through at once, and in the ſhort ſpace of two or three ſeconds, the ſkin, the cellular ſubſtance, the neck of the bladder, the muſcles, and proſtate gland; advantages, which entitle this method to be preferred above all others, particularly as they have already been proved by experience. Citizen Guerin has ſeveral times performed this operation with great ſucceſs, and likewiſe Treyerau of Bourdeaux, and Petit of Lyons, who obſerved, that the wounds healed in a much ſhorter time, and without any fever. Peletan has alſo tried it with ſucceſs in the Hotel-Dieu.

## Figures and Tables

**Fig. 3. Fig. 2. Fig. 1 f1:**